# Spinal cord injury–induced overflow incontinence reshapes the activin–follistatin–inhibin axis in mouse bladder and kidney

**DOI:** 10.3389/fmolb.2026.1752395

**Published:** 2026-02-18

**Authors:** Yucheng Shen, Shengkai Yang, Hai Zhou, Yongkun Zhu, Zhong Wang, Weimin Jiang

**Affiliations:** 1 The First Affiliated Hospital of Soochow University, Suzhou, China; 2 Binhai County People’s Hospital, Yancheng, China; 3 Southeast University School of Medicine, Nanjing, China

**Keywords:** activin–follistatin–inhibin axis, gene expression, immunofluorescence, kidney, neurogenic bladder, spinal cord injury, western blot

## Abstract

**Background:**

Spinal cord injury is a leading cause of neurogenic bladder and upper urinary tract deterioration, yet the molecular remodeling of epithelial-stromal signaling axes in this context remains incompletely defined. The activin-follistatin-inhibin axis, a branch of the transforming growth factor-β superfamily, has been implicated in tissue repair, inflammation, and fibrosis, but its behavior in the lower urinary tract after SCI is unknown.

**Methods:**

A standardized T9–T10 contusion SCI model was established in male C57BL/6J mice. Bladder dysfunction was assessed using the voiding spot assay. Gross morphology and organ weights of the bladder and kidneys were recorded. Quantitative RT–PCR was used to profile mRNA expression of 11 AFI-axis genes in bladder and kidney. Western blotting was performed for selected proteins, and immunofluorescence was used to map the spatial distribution of FST, INHA and INHBE in bladder and INHA, INHBB and INHBE in kidney.

**Results:**

SCI mice displayed numerous irregular urine spots with leakage/tailing on the voiding spot assay and an increased voided area, accompanied by marked bladder distension and increased bladder and kidney weights compared with sham controls, suggesting compromised voiding efficiency and impaired bladder emptying. In the bladder, *Fst* mRNA was robustly upregulated, whereas *Inha*, *Inhbc*, and *Inhbe* were significantly downregulated; in the kidney, *Acvr1b* and *Inhbb* were increased, while *Inha*, *Inhbc* and *Inhbe* were decreased. At the protein level, bladder FST was increased and INHA and INHBC were reduced, whereas in the kidney INHA and INHBE were decreased and INHBB was increased, with no change in INHBC. Immunofluorescence showed enhanced subepithelial and stromal FST staining in SCI bladders, redistribution of INHA toward the stroma, and increased stromal INHBE signals, while INHA, INHBB, and INHBE remained broadly distributed across renal compartments with modest intensity shifts.

**Conclusion:**

SCI reshapes the AFI axis in both bladder and kidney, characterized by coordinated but regionally distinct alterations in FST, INHA, INHBE, and INHBB expression. These findings extend the concept that the AFI axis contributes to urothelial–stromal homeostasis and suggest that AFI-axis remodeling is part of the molecular signature of neurogenic bladder and associated renal adaptation after SCI. These observational findings nominate the AFI axis as a priority pathway for future mechanistic and interventional evaluation, with therapeutic potential to be determined using objective functional endpoints.

## 1 Introduction

Spinal cord injury (SCI) is one of the leading causes of bladder dysfunction, with overflow incontinence representing a hallmark clinical manifestation (Burns et al., 2001; Almajid et al., 2017). Neurogenic bladder secondary to SCI not only compromises the quality of life but also predisposes patients to recurrent urinary tract infections and progressive renal impairment (Almajid et al., 2002). However, most studies on post-SCI bladder dysfunction have focused on neural reflex circuits and detrusor electrophysiology, whereas alterations in local epithelial–stromal signaling networks remain largely unexplored (Ryu et al., 2018).

The activin–follistatin–inhibin (AFI) axis, a branch of the transforming growth factor-β (TGF-β) superfamily, plays pivotal roles in embryonic development, tissue repair, inflammation, fibrosis, and tumorigenesis (Hedger and de Kretser, 2013). The AFI axis comprises three functional components: (i) a ligand family (*Inha*, *Inhba*, *Inhbb*, *Inhbc*, and *Inhbe*) encoding distinct activin and inhibin isoforms that modulate cellular proliferation and differentiation; inhibins are heterodimers composed of inhibin α (INHA) and a β subunit (INHBB, INHBC, or INHBE); (ii) a receptor family (*Acvr1*, *Acvr1b*, *Acvr1c*, *Acvr2a*, and *Acvr2b*) that mediates ligand-induced small mother against decapentaplegic (SMAD) signaling; and (iii) the binding protein follistatin (*Fst*), which antagonizes activin signaling by competitive sequestration, thereby maintaining signaling homeostasis. Together, these elements form an integrated regulatory network of the AFI axis (De Jong, 1988; Barrero et al., 2009; Tsuchida et al., 2009). Previous studies have revealed a distinctive spatial organization of the AFI axis in the normal bladder: Activin A receptor type 1B (ACVR1B) and Activin A receptor type 2B (ACVR2B) are localized to the basolateral membranes of urothelial cells, activin-positive cells are predominantly distributed within the stromal compartment, and FST forms a thin, basement membrane–like barrier separating the two. This spatial segregation suggests that the AFI axis contributes to bladder homeostasis through fine-tuned paracrine regulation (Mao et al., 2025). Chronic bladder distension, inflammation, and tissue remodeling following SCI may disrupt this epithelial-stromal barrier, facilitating aberrant activin-receptor interactions and pathological activation of downstream signaling cascades. The lower urinary tract dysfunction resulting from SCI is frequently accompanied by progressive deterioration of the upper urinary tract, including kidney injury. While bladder dysfunction in neurogenic bladder has been extensively studied, there is increasing recognition that kidney dysfunction, especially secondary to high intravesical pressure and urinary retention, plays a critical role in the long-term morbidity of SCI patients (Wang et al., 2024). The AFI axis has been implicated in various pathophysiological processes, including inflammation and fibrosis, in multiple tissues. Given the evidence linking AFI axis components to kidney injury and fibrosis (Tsai et al., 2024; Wang et al., 2025), we aimed to profile the AFI axis in both the bladder and kidney to assess whether similar molecular alterations occur in the upper urinary tract.

Here, we established a standardized mouse SCI model and characterized SCI-associated voiding behavior using the voiding spot assay, together with gross bladder morphology and organ weight measurements. We subsequently profiled mRNA and protein expression of key AFI-axis components in the bladder and kidney, coupled with immunofluorescence-based spatial mapping. These analyses aimed to delineate how SCI remodels the AFI axis within the urinary tract, thereby providing molecular insight into the pathogenesis of neurogenic bladder and providing a foundation for future mechanistic and interventional validation.

## 2 Materials and methods

### 2.1 Animals and groups

Male C57BL/6J mice (8–12 weeks old) were purchased from Vitonlihua Laboratory Animal Technology Co., Ltd. (Beijing, China). A total of 6 male mice were used and randomly assigned to the SCI group (n = 3) or the sham group (n = 3). In this study, the sample size n = 3 explicitly refers to biological replicates, with each unit representing an individual mouse to capture natural biological variability. Mice were housed under specific pathogen-free (SPF) conditions at 25 °C on a 12 h light/12 h dark cycle with *ad libitum* access to food and water. After completion of the VSA on day 7 post-SCI, mice were immediately anesthetized with 5% isoflurane via inhalation for induction (maintained at 1.5%–2% isoflurane during subsequent procedures to ensure adequate anesthesia depth, verified by absence of pedal and corneal reflexes). Mice were then euthanized by inhalation of excess carbon dioxide using a gradually filling chamber at a flow rate of 20% volume/min. The bladder and kidneys were rapidly harvested immediately after euthanasia, and residual urine was gently expressed from the bladder. These same tissues were processed for all downstream molecular assays (qRT-PCR, Western blotting, and immunofluorescence), ensuring that all readouts corresponded to a single defined post-injury time point. The bladder and kidneys were weighed on a precision balance (BSA224S-CW, Sartorius), recorded in grams (g), and processed according to predefined protocols. All animal procedures were approved by the Animal Ethics Committee of Southeast University (approval No.: SEU-IACUC-20250325003) and were conducted in accordance with relevant institutional and national guidelines.

### 2.2 Spinal cord injury model establishment

A standardized contusion model of spinal cord injury was established. Mice were anesthetized with isoflurane (induction 5%, maintenance 1%–2%), placed in the prone position, and stabilized with a cervical collar to restrict head and neck movement. Following a T9–T10 laminectomy, the spinal cord was exposed and subjected to a moderate contusion using a precision impactor (Model 68099II, RWD Life Science, Shenzhen, China) with parameters set to 1 m/s impact velocity, 0.6 mm depth, and 0.5 s dwell time. The incision was closed in layers, and mice recovered under a heating lamp to maintain body temperature. Sham mice underwent identical anesthesia, laminectomy, and exposure without contusion, serving as surgical controls. Both groups were monitored postoperatively and allowed to recover.

#### 2.2.1 Voiding spot assay

Voiding spot assays (VSA) were performed on post-injury days 6–7, between 09:00 and 13:00 (4 h total). Individual mice were placed in metabolic cages lined with filter paper (21.7 cm × 13.9 cm × 1 mm) to collect urine. Food was provided during the test; water was withheld. At the end of the session, filter papers were removed and imaged under 365 nm ultraviolet illumination using a UV analyzer. Images were captured with a digital single-lens reflex camera and saved in JPG format. Processing was performed in ImageJ: images were converted to 8-bit grayscale, a fixed grayscale threshold was applied, and urine spots on the filter paper were identified.

### 2.3 Quantitative real-time PCR analysis

Total RNA from bladder and kidney tissues was extracted using the RNAeasy™ RNA Isolation Kit (Beyotime Biotechnology, Shanghai, China) following the manufacturer’s instructions. RNA concentration and purity were assessed with a NanoDrop™ 2000 spectrophotometer (Thermo Fisher Scientific, USA). cDNA was synthesized using the SweScript RT II First Strand cDNA Synthesis Kit (Servicebio, Wuhan, China). qPCR was performed with SYBR Green qPCR Master Mix (Servicebio, Wuhan, China). Relative expression of target genes (*Acvr1*, *Acvr1b*, *Acvr1c*, *Acvr2a*, *Acvr2b*, *Fst*, *Inha*, *Inhba*, *Inhbb*, *Inhbc*, *Inhbe*) was normalized to β-actin. Fold changes were calculated using the 2^–ΔΔCt^ method. To ensure technical precision, each biological sample (n = 3 mice per group) was analyzed in technical triplicates. The cycle threshold values from these triplicates were averaged to provide a single value for each biological replicate. All samples were run in triplicate, and results are reported as fold change relative to the sham group. Primer sequences are provided in Table 1.

**TABLE 1:** Primer sequences for RT-qPCR analysis of AFI axis genes.

Gene	Forward primer (5'→ 3′)	Reverse primer (5'→ 3′)
Acvr1	CCATTGAAGGGCTCATCACCAC	CCGTTCTCTGTACCAGGAAAGG
Acvr1b	ACGAAGATGCAATTCTGGAGG	TCTTTCCCATCACTCGCAAG
Acvr1c	GCTGACATCTATTCGGTGGG	TTTGGGAGATTTGGTCGGAG
Acvr2a	GCGACATTGTTTTGCTACCTG	ACACATATTGCCCTCACAGC
Acvr2b	AAGCCTTCTATTGCCCACAG	TCAAACCGAACAGCCAGG
Fst	TGTAATCGGATTTGCCCAGAG	CACACTGGATATCTTCACAGGAC
Inha	CTAGACAGAAAGGGCACAGG	AGGGTCAACAGCAAAAGGAG
Inhba	ATCACCTTTGCCGAGTCAG	TGCTGAAATAGACGGATGGTG
Inhbb	TCCGAGATCATCAGCTTTGC	GGGAGCAGTTTCAGGTACAG
Inhbc	TGACAGGGACAGCAACATTG	GGACAGAAGTGGGAACAGAG
Inhbe	CTGCTTCTGTATCCTCTTTGGG	CTTCTACTCTGCACCCACAC

### 2.4 Western blot analysis

Proteins from bladder and kidney tissues were extracted with RIPA lysis buffer (Servicebio, Wuhan, China). Protein concentrations were measured using a BCA Protein Assay Kit (Keygen Biotech, Nanjing, China). Equal amounts of protein were heated at 100 °C for 5 min, resolved by 10% SDS-PAGE, and transferred to PVDF membranes. Membranes were blocked with 5% non-fat milk for 1 h at room temperature, washed with TBST, incubated with primary antibodies at 4 °C overnight, washed three times, and then incubated with the appropriate secondary antibodies for 1 h at room temperature. After three additional TBST washes, signals were developed with an automated exposure system, and band intensities were quantified using ImageJ. To account for potential variations in protein loading, the optical density of each target protein band was normalized to the intensity of its corresponding β-actin loading control (expressed as the Target/β-actin ratio). Blots were cropped to display representative target bands and the corresponding loading controls. Each lane in the Western blot represents an independent protein extract derived from a single mouse (n = 3 biological replicates). Anti-β-actin was used as a loading control. The primary antibodies used in this study are listed in Table 2.

**TABLE 2:** Antibody list used in the study.

Antibody name	Manufacturer	Catalog number	Application, dilution
Fst	Proteintech	60060-1-Ig	WB, 1:2000; IF, 1:400
Inha	Proteintech	27331-1-AP	WB, 1:2000; IF, 1:200
Inhbb	Thermofisher	MA5-53081	WB, 1:2000
Inhbb	BIOSS	bs-1825R	IF, 1:100
Inhbc	Thermofisher	PA5-103143	WB, 1:2000
Inhbe	BIOSS	bs-16658R	WB, 1:1000; IF, 1:100
β-actin	Proteintech	66009-1-Ig	WB, 1:10000

### 2.5 Immunofluorescence analysis

Freshly excised bladder and kidney tissues were fixed for 2 h at room temperature in 4% paraformaldehyde (PFA) prepared in 100 mM sodium cacodylate buffer (pH 7.4), then embedded in optimal cutting temperature (OCT) compound. Cryosections (5 µm) were prepared and incubated with primary antibodies at 4 °C overnight, followed by fluorophore-conjugated secondary antibodies for 1 h at room temperature. Nuclei were counterstained with DAPI. Images were acquired on a BX60 Olympus fluorescence microscope using cellSens v4.3 software with a 40×/0.75 objective and saved as TIFF files. Fluorescence channels captured at different wavelengths were merged, and image contrast was adjusted in Adobe Photoshop. For semi-quantitative analysis of the mean fluorescence intensity (MFI), the integrated density of specific fluorescent signals was measured using ImageJ software. Briefly, single-channel fluorescent images were imported, and the region of interest (ROI) was manually delineated to cover the target tissue/cellular compartments in a blinded manner; the background fluorescence was subtracted by measuring the MFI in the adjacent non-specific tissue areas without positive staining. The MFI of the target signals was then normalized to the nuclear DAPI fluorescence intensity of the same ROI to eliminate potential variations from section thickness and sampling differences. At least 5 non-overlapping visual fields were randomly selected from each section, with three biological replicates per experimental group. Antibody details are provided in Table 2.

### 2.6 Statistical analysis

All data are presented as the mean ± standard deviation (SD) of n = 3 independent biological replicates (individual animals). Comparisons between two groups were performed using two-tailed Student’s t-tests. To account for the multiple simultaneous hypotheses tested across different genes and organs, P values were adjusted using Šídák’s multiple comparisons test. A P value <0.05 was considered statistically significant. All analyses were conducted using GraphPad Prism software (version 10.3.0, California, USA).

## 3 Results

### 3.1 Validation of spinal cord injury model and kidney/bladder weight analysis

**FIGURE 1:**
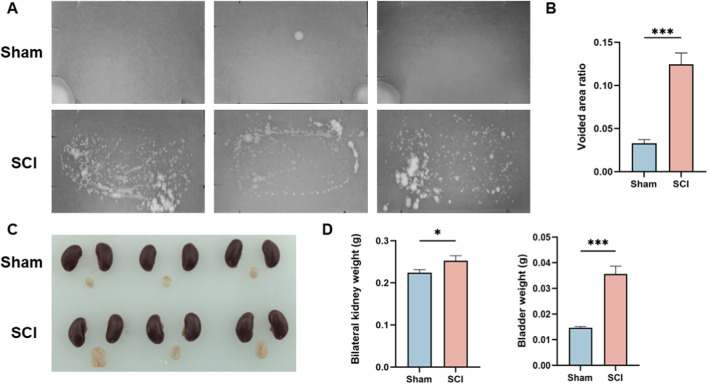
Functional and Morphological Validation of the Spinal Cord Injury Model. **(A)** Representative voiding spot assay images from Sham and SCI groups. **(B)** Quantification of the percentage of voiding spot area relative to total filter paper area. **(C)** Macroscopic observation of bladder and kidney morphology in Sham and SCI groups, with the bladder in the SCI group showing significant enlargement and distension. Kidneys are shown as representative appearance only; no dedicated gross assessment of renal morphology (including hydronephrosis/upper-tract dilation) was performed. **(D)** Quantification of the bilateral kidney and bladder weights, with both bladder and kidney weights significantly increased in the SCI group compared to the Sham group. Data are presented as mean ± standard deviation (mean ± SD). (n = 3) N.S. P > 0.05, not significant; *P < 0.05.

Bladder dysfunction in SCI mice was assessed using the voiding spot assay. As shown in Figure 1A, SCI mice exhibited numerous irregularly shaped urine spots, accompanied by leakage and tailing. In contrast, sham mice displayed only a few well-defined, round spots, without evidence of leakage or tailing. Quantitative analysis of the urine spot area revealed that SCI mice had a significantly higher percentage of voided area relative to the total filter area compared to sham controls (Figure 1B).

In addition to functional assessment, macroscopic examination of the bladder and kidneys was performed (Figure 1C). The bladder in SCI mice was notably enlarged and distended. Kidneys are included for visual reference; however, no dedicated renal morphological assessment (e.g., grading of hydronephrosis or upper-tract dilation) was performed in this study. Quantitative analysis (Figure 1D) showed that both bladder and bilateral kidney weights were significantly higher in SCI mice than in sham mice.

### 3.2 Gene expression of AFI axis in bladder and kidney

Quantitative RT-PCR was performed to assess mRNA expression of 11 AFI-axis–related genes, including ligands (*Inha*, *Inhba*, *Inhbb*, *Inhbc*, and *Inhbe*), receptors (*Acvr1*, *Acvr1b*, *Acvr1c*, *Acvr2a*, and *Acvr2b*) and *Fst*. In the bladder, *Inha*, *Inhbc*, and *Inhbe* showed relatively high expression, while *Inhba*, *Inhbb*, and receptor genes such as *Acvr1* and *Acvr1c* were expressed at low or undetectable levels. Similarly, in the kidney, *Acvr1c* and *Fst* were expressed at very low levels.

**FIGURE 2:**
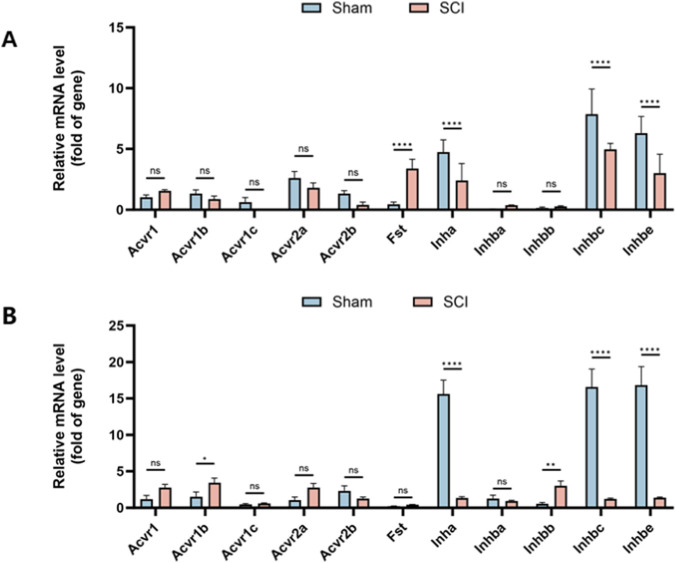
Expression Profile of AFI Axis Genes in Bladder and Kidney from Sham and SCI Groups. Quantitative RT-PCR was used to measure mRNA expression of 11 AFI axis genes in bladder **(A)** and kidney **(B)** tissues. **(A)** In the bladder, Fst expression was significantly increased, while Inha, Inhbc, and Inhbe were significantly decreased, with no significant changes in other genes. **(B)** In the kidney, Acvr1b and Inhbb were upregulated, while Inha, Inhbc, and Inhbe were significantly downregulated, with no significant changes in other genes. (n = 3) N.S. P > 0.05, not significant; *P < 0.05, **P < 0.01, ***P < 0.001, ****P < 0.0001.

After SCI, *Fst* expression in the bladder was markedly increased (adjusted P < 0.0001, fold change = 7.17), whereas *Inha*, *Inhbc*, and *Inhbe* were significantly decreased compared with sham controls (adjusted P < 0.0001; fold change = 0.51, 0.63, and 0.48, respectively). No significant differences were observed in *Acvr1*, *Acvr1b*, *Acvr1c*, *Acvr2a*, *Acvr2b*, *Inhba*, or *Inhbb* (all adjusted P ≥ 0.3348) (Figure 2A).

In the kidney, *Acvr1b* and *Inhbb* were significantly upregulated (adjusted P = 0.0103 and 0.0014; fold change = 2.24 and 5.44, respectively), while *Inha*, *Inhbc*, and *Inhbe* were markedly downregulated (all adjusted P < 0.0001; fold change = 0.086, 0.075, and 0.083, respectively); other genes showed no significant change (all adjusted P ≥ 0.0583) (Figure 2B).

### 3.3 Protein expression of AFI axis in bladder and kidney

**FIGURE 3:**
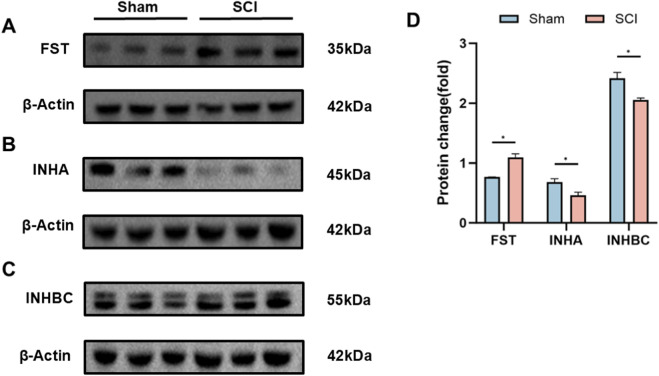
Protein Expression of AFI Axis Members in Bladder from Sham and SCI Groups. **(A–C)** Western blot analysis of FST **(A)**, INHA **(B)**, and INHBC **(C)** protein expression in mouse bladder. Target bands were identified by comparison with molecular weight markers (FST: ∼35 kDa; INHA: ∼45 kDa; INHBC: ∼55 kDa; β-actin: ∼42 kDa). **(D)** Quantification of FST, INHA, and INHBC protein expression between SCI and Sham groups. β-actin was utilized as the loading control for each independent blot. Bar graphs display the mean ± SD of target protein intensities normalized to β-actin. (n = 3) N.S. P > 0.05, not significant; *P < 0.05.

Western blot analysis was conducted to evaluate protein expression of selected AFI components in the bladder and kidney. In the bladder, FST, INHA, and INHBC were examined. Compared with sham controls, SCI mice exhibited significantly elevated FST expression (adjusted P = 0.0017, fold change = 1.43) and markedly reduced INHA and INHBC levels (adjusted P = 0.0077 for both; fold change = 0.68 and 0.85, respectively) (Figure 3). INHBE protein could not be reliably detected in bladder samples by Western blot under our experimental conditions and was therefore not included in the bladder Western blot panel.

**FIGURE 4:**
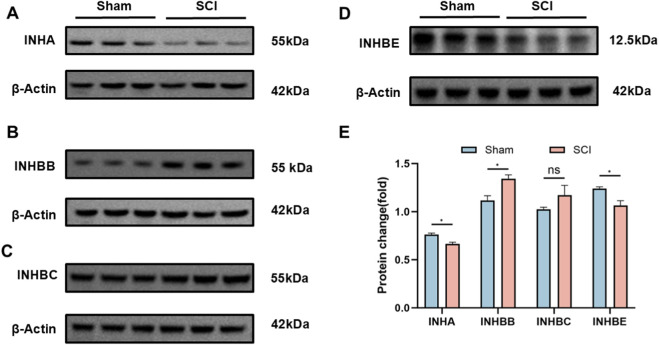
Protein Expression of AFI Axis Members in Kidney from Sham and SCI Groups. **(A–D)** Western blot analysis of INHA **(A)**, INHBB **(B)**, INHBC **(C)**, and INHBE **(D)** protein expression in mouse kidney. Target bands were identified by comparison with molecular weight markers (INHA: ∼55 kDa; INHBB: ∼55 kDa; INHBC: ∼55 kDa; INHBE: ∼12.5 kDa; β-actin: ∼42 kDa). **(E)** Quantification of INHA, INHBB, INHBC, and INHBE protein expression between SCI and Sham groups. β-actin was utilized as the loading control for each independent blot. Bar graphs display the mean ± SD of target protein intensities normalized to β-actin. (n = 3) N.S. P > 0.05, not significant; *P < 0.05.

In the kidney, INHA, INHBB, INHBC, and INHBE were analyzed. SCI mice showed reduced INHA and INHBE expression (adjusted P = 0.0055 and 0.0099; fold change = 0.87 and 0.86), increased INHBB expression (adjusted P = 0.0099; fold change = 1.20), and no significant difference in INHBC levels (adjusted P = 0.0654; fold change = 1.14) (Figure 4). These protein-level changes were consistent with the transcriptional results.

### 3.4 Immunofluorescence analysis of AFI axis proteins in the bladder and kidney

**FIGURE 5:**
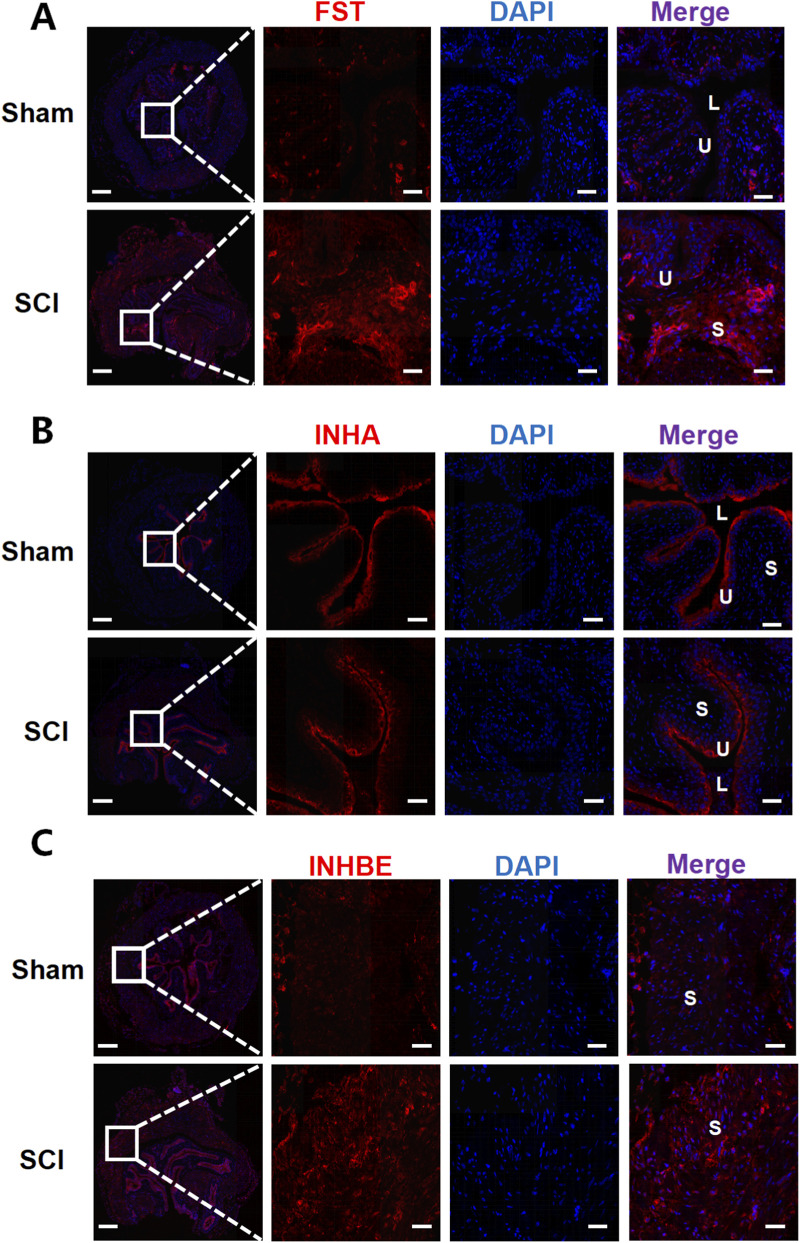
Immunofluorescence analysis of AFI-axis proteins in the bladder. Representative immunofluorescence images of bladder sections from Sham and SCI mice stained for FST **(A)**, INHA **(B)**, and INHBE **(C)** (red), with nuclei counterstained with DAPI (blue). For each marker, low-magnification overview images (left) are shown with a boxed region; dashed lines indicate the corresponding area displayed at higher magnification in the adjacent single-channel and merged images. In merged images, U denotes urothelium, S denotes stroma, and L denotes bladder lumen. Scale bars: 200 μm (overview images) and 50 μm (magnified images).

Immunofluorescence staining was used to examine the spatial distribution and expression changes of key AFI axis members in the bladder and kidney following SCI. In the bladder, FST, INHA, and INHBE exhibited distinct localization patterns (Figure 5). FST was primarily localized to the subepithelial and stromal regions, and while the fluorescence intensity of FST was significantly increased in the SCI group compared to the sham group, the overall expression pattern did not change. INHA exhibited strong staining in the epithelium and subepithelial regions in the sham group. Following SCI, the staining intensity in these regions showed no significant change, whereas its expression in the interstitial region was increased. INHBE immunoreactivity was broadly detected in both the urothelial and stromal compartments in sham bladders; following SCI, INHBE fluorescence increased in the urothelium and was more prominently enhanced within the stroma.

**FIGURE 6:**
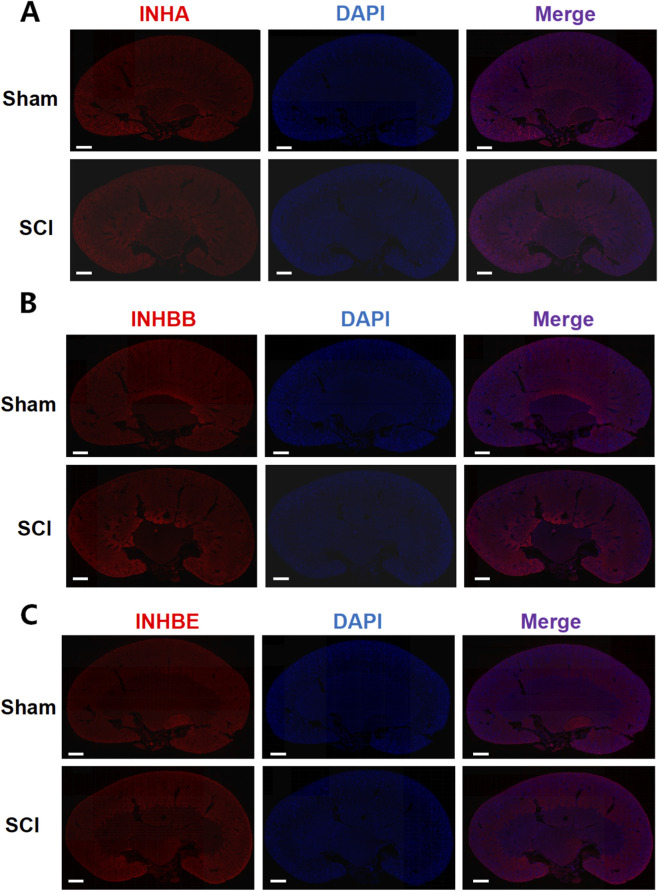
Immunofluorescence analysis of AFI-axis proteins in the kidney. Representative immunofluorescence images of kidney sections from Sham and SCI mice stained for INHA **(A)**, INHBB **(B)**, and INHBE **(C)** (red), with nuclei counterstained with DAPI (blue). For each marker, single-channel images and the corresponding merged images are shown to illustrate the overall spatial distribution of the target proteins within the kidney. Scale bars: 625 μm.

In the kidney, INHA, INHBB, and INHBE were broadly distributed across the renal tubules, cortex, and other regions in both the sham and SCI groups (Figure 6). INHA fluorescence intensity slightly decreased in SCI mice, whereas INHBB exhibited a mild increase in fluorescence intensity. No significant change in INHBE fluorescence intensity was observed between the two groups. These observations were supported by semi-quantitative ROI-based MFI analysis, with statistical plots provided in Supplementary Figures S1, S2.

## 4 Discussion

This study aimed to elucidate how spinal cord injury reshapes the expression of the activin-follistatin-inhibin axis in the bladder and kidney, thereby advancing our understanding of the molecular underpinnings of neurogenic bladder dysfunction. Our findings demonstrate that SCI induces marked alterations in key AFI-axis components, most notably a robust upregulation of FST in the bladder and a significant reduction of INHA at both the mRNA and protein levels. For INHBE, although its mRNA expression in the bladder was decreased after SCI, no specific band could be detected by Western blot under our experimental conditions, precluding reliable assessment of overall protein abundance. By contrast, immunofluorescence revealed enhanced INHBE staining in the stromal compartment of SCI bladders. In the kidney, the AFI axis exhibited a distinct remodeling pattern: *Inhbb* expression was increased, whereas *Inha* and *Inhbe* were reduced at both the transcript and protein levels, with INHBE immunofluorescent staining showing no overt difference between sham and SCI groups. Together, these observations provide new molecular clues to how SCI perturbs bladder function and impacts the upper urinary tract, and they identify AFI-axis remodeling as a candidate molecular signature and highlight AFI-axis components as mechanistic nodes and/or biomarkers worthy of functional validation in neurogenic bladder.

One of the principal findings of this work is the pronounced upregulation of FST in the bladder following SCI, accompanied by preservation of its characteristic suburothelial and stromal localization. As a high-affinity activin-binding protein, follistatin constrains the activity of TGF-β family ligands within the local microenvironment by limiting ligand availability to their receptors (Hedger and de Kretser, 2013). In the present study, FST was increased at the mRNA, protein, and immunofluorescence levels in SCI bladders, without an apparent reconfiguration of its spatial distribution. This pattern supports a model in which FST responds to chronic distension, inflammation, and matrix remodeling primarily through quantitative amplification rather than positional redistribution. Importantly, fibrosis- and inflammation-related tissue readouts (e.g., ECM deposition or inflammatory cytokine markers) were not directly quantified in this study, and thus these interpretations remain inferential. Such upregulation may represent a protective attempt to buffer excessive activin activity, yet under certain conditions, excessive sequestration of activin could also impair appropriate repair responses or epithelial differentiation and thereby contribute to maladaptive remodeling.

Within the bladder, the concordant decrease of INHA at both transcript and protein levels adds a second layer of imbalance to the AFI axis. INHA encodes the α subunit of inhibin, which antagonizes activin signaling by competitively engaging activin receptors (De Jong, 1988). Thus, the overall reduction of INHA suggests a shift of the activin–inhibin equilibrium toward diminished inhibin-mediated antagonism. Immunofluorescence refines this picture further: in sham bladders, INHA is predominantly localized to the urothelium and suburothelial region, whereas in SCI bladders, staining intensity in these layers remains largely unchanged but shows a modest increase in the stroma. This combination of “global reduction with relative stromal enrichment” indicates that SCI not only attenuates the overall inhibitory capacity of inhibin toward activin, but also remodels the AFI-axis architecture at the epithelial-stromal interface. Such remodeling may alter how activin signals are integrated among urothelial cells, suburothelial myofibroblasts, and resident immune cells, with potential consequences for barrier integrity, matrix turnover, and the chronic inflammatory milieu characteristic of neurogenic bladder.

The behavior of INHBE in the bladder further underscores the complexity of AFI-axis regulation across the gene-protein-tissue continuum. At the transcript level, Inhbe was significantly downregulated after SCI; however, under our experimental conditions, immunoblotting did not yield a resolvable INHBE-specific band, consistent with very low abundance in bladder homogenates and/or limited antibody performance for quantitative Western blotting. To obtain spatially resolved information, we therefore performed immunofluorescence, which revealed increased INHBE signal after SCI in both the urothelium and the stroma, with a more prominent enhancement in the stromal compartment. This divergence between a homogenate-level mRNA reduction and compartment-enriched fluorescence gains is best interpreted as platform- and compartment-dependent, rather than as a single-layer contradiction. qRT-PCR reports an average transcript signal pooled from heterogeneous tissue fractions, whereas immunofluorescence captures antigen signals within defined microanatomical compartments; thus, SCI-associated shifts in tissue composition/cellular sources, together with post-transcriptional control, differential protein stability across microenvironments, and methodological differences between bulk extraction and *in situ* detection, could jointly contribute to the observed pattern. Importantly, these observations do not negate the consistent transcript-level decrease, but instead highlight that spatial redistribution and local enrichment can occur despite an overall reduction in transcript abundance, and that conventional bulk assays may under-represent such compartment-specific modulation. Future compartment-resolved approaches (e.g., urothelium-stroma separation, laser-capture microdissection, or spatial transcriptomics) will be valuable for reconciling transcript- and tissue-level readouts.

In the kidney, the AFI axis exhibited a remodeling pattern distinct from that observed in the bladder. Quantitative RT–PCR revealed upregulation of *Acvr1b* and *Inhbb*, alongside downregulation of *Inha*, *Inhbc*, and *Inhbe*. Consistent with these findings, Western blot analysis confirmed increased INHBB protein levels and decreased INHA and INHBE, while total INHBC protein remained largely unchanged. Immunofluorescence demonstrated that INHA, INHBB, and INHBE were broadly distributed throughout the renal parenchyma in both sham and SCI groups. Following SCI, the overall staining pattern was largely preserved, with only a reduction in INHA signal intensity, a modest enhancement of INHBB, and essentially stable INHBE staining. These observations suggest that, in contrast to the more pronounced spatial remodeling seen in the bladder, the renal response to SCI is mediated primarily through fine-tuning of AFI-axis expression levels within a pre-existing anatomical framework. The combination of INHBB upregulation with INHA and INHBE downregulation may shift activin family signaling toward a more pro-inflammatory or pro-fibrotic profile, in line with previous reports linking activin family members to renal injury and fibrosis (Wijayarathna and de Kretser, 2016; Bian et al., 2025; Nagayama et al., 2025).

In interpreting the renal findings, we emphasize that kidney alterations observed after SCI may predominantly reflect secondary consequences of lower urinary tract dysfunction—such as sustained urinary retention and elevated intravesical pressure with potential upper-tract reflux—rather than a direct effect of the thoracic lesion *per se*. Accordingly, the renal AFI-axis changes detected here are discussed in the context of post-SCI voiding dysfunction imposing pressure/retention-related stress on the upper urinary tract. Our data therefore support an association between SCI-induced bladder dysfunction and renal AFI-axis remodeling, while the direct versus secondary nature of these renal alterations remains to be resolved.

Several methodological considerations must be taken into account when interpreting these renal findings. First, although *Acvr1b* mRNA levels were increased after SCI, its basal expression was low, and the lack of a highly specific and sensitive primary antibody precluded reliable quantification of ACVR1B protein by Western blot. As a result, conclusions regarding activin receptor remodeling rest predominantly on transcriptional data and are not supported by direct protein-level validation. Second, while INHBC could be readily detected and quantified by Western blot in both bladder and kidney, we were unable to obtain antibodies that yielded specific, interpretable immunofluorescent signals in tissue sections, preventing assessment of its spatial redistribution during SCI. These technical limitations do not negate the mRNA and bulk protein trends we observed, but they do constrain inferences about AFI-axis reorganization at the microanatomical level and warrant caution when extrapolating these molecular changes to specific pathophysiological processes.

Our study provides a novel molecular perspective on the pathophysiology of neurogenic bladder following SCI. Previous work has focused predominantly on neural circuit reorganization, detrusor overactivity, and growth factor-related pathways, such as the proNGF/p75 axis in the SCI bladder (de Groat, 1998; Ryu et al., 2018; Peyronnet et al., 2019; Ma et al., 2023),whereas the AFI axis has been studied mainly in the context of reproductive endocrinology, inflammation, and pregnancy-related disorders (Perakakis et al., 2018; Bouzoni et al., 2020; Bouzoni et al., 2022). Building on prior mapping of AFI-axis distribution in the normal bladder,the present study is, to our knowledge, the first to demonstrate at a systems level that SCI-induced neurogenic bladder is accompanied not only by local remodeling of AFI-axis components within the bladder wall, but also by parallel alterations in upper urinary tract structures such as the kidney (Mao et al., 2025). By integrating data from voiding spot assays, organ weight measurements, gross morphology, qRT-PCR, Western blotting, and spatially resolved immunofluorescence, we delineate a cross-organ, multi-node pattern of AFI-axis reprogramming that would be difficult to capture using any single methodological approach.

These findings have several important implications. Neurogenic bladder after SCI is fundamentally initiated by disruption of afferent and efferent neural pathways that govern bladder sensation and detrusor–sphincter coordination. Within this well-established neurogenic framework, progressive bladder-wall remodeling—particularly fibrosis and epithelial-stromal reprogramming—can accumulate and contribute to persistent dysfunction and heightened upper-tract vulnerability. Our data therefore position AFI-axis remodeling as a molecular correlate of bladder and renal tissue remodeling that accompanies SCI-induced voiding dysfunction, rather than as an alternative explanation that diminishes the central role of neural injury. Although the present study is descriptive and does not directly interrogate cell-specific mechanisms, the observed, compartment-resolved shifts in AFI-axis members are consistent with prior evidence linking activin/inhibin/follistatin signaling to repair and fibrotic remodeling, and motivate future functional studies to define how AFI-axis perturbation interfaces with post-SCI fibrosis and long-term bladder deterioration. In line with this broader framework, activin signaling—via the TGF-β superfamily and SMAD2/3-related programs—has been functionally implicated in fibrotic remodeling in multiple organ systems. In the bladder, partial inhibition of the activin type I receptor ALK4 (ACVR1B) has been reported to attenuate fibrosis in experimental obstruction models, supporting a profibrotic ALK4–SMAD2/3 axis (Wang et al., 2021). In the kidney, activin A has been linked to inflammatory and profibrotic injury responses, and antagonizing activin A signaling via follistatin has been shown to reduce renal fibrosis and inflammation in disease models (Bian et al., 2025). However, because we did not directly manipulate AFI-axis components or quantify fibrosis/inflammation markers in the current dataset, these mechanistic links remain hypothesis-generating and require targeted validation in future studies. From a translational perspective, AFI-axis constituents may serve as candidate molecular markers for disease stratification, progression monitoring, and therapeutic response. The emerging evidence that targeting FST or activin-receptor interactions can be beneficial in other fibrotic and inflammatory conditions suggests that judicious modulation of this axis in neurogenic bladder could complement existing neuromodulatory or surgical interventions (Tsuchida et al., 2009).

At the same time, several limitations of this study warrant cautious interpretation. First, the present dataset was generated in a single mouse strain and sex with modest group sizes (n = 3 per group) and at a single post-injury endpoint, which limits statistical power and may not capture sex-dependent effects or temporal dynamics of AFI-axis remodeling. Although the post-injury interval and endpoint are specified in the Methods, we emphasize here that the current study represents a focused snapshot rather than a longitudinal trajectory. In this context, we used a male cohort to provide an initial, internally consistent molecular snapshot of AFI-axis remodeling after SCI and to align with the male predominance reported in traumatic SCI at the population level (World Health Organization, 2024); nevertheless, murine lower urinary tract function exhibits sex-dependent voiding–sphincter coordination, and recent work using cystometry combined with EUS-EMG has demonstrated distinct sex-specific patterns in spinal-intact animals and after SCI with corresponding differences in voiding efficiency (Hashimoto et al., 2024). Accordingly, the functional interpretation and AFI-axis remodeling reported here should be considered male-specific and not assumed to generalize across sexes. Future studies with larger cohorts, inclusion of female mice, and multiple post-injury time points will be essential to define robustness, inter-individual variability, and the evolution of AFI-axis changes across the acute-to-chronic transition. Furthermore, our work is primarily descriptive and correlative: we did not directly manipulate AFI-axis components—for example, via gene knockdown or pharmacological blockade—to establish causal links between AFI reprogramming and functional changes in the bladder or kidney. In addition, because fibrosis/inflammation markers in bladder and kidney (e.g., collagen/fibronectin/α-SMA or inflammatory cytokine and immune-cell infiltration readouts) were not assessed, the functional relevance of AFI-axis remodeling to these downstream pathophysiological processes should be interpreted with caution. Because vesicoureteral reflux/hydronephrosis, urodynamic parameters, and renal functional indices were not directly assessed, we cannot discriminate whether the renal changes are direct consequences of SCI or secondary to bladder dysfunction. And the kidney assessment was limited to gross inspection, and dedicated renal pathology readouts (e.g., histology for subtle hydronephrosis, tubular injury, or fibrosis) were not performed; therefore, microscopic renal changes cannot be excluded. In addition, technical constraints prevented detection of INHBE protein in the bladder by Western blot, reliable assessment of ACVR1B protein in the kidney, and immunofluorescent validation of INHBC localization in either organ. Functional evaluation of bladder performance was largely based on voiding spot assays and organ weights, and although the voiding spot assay provides a practical, noninvasive readout of spontaneous voiding behavior, it does not provide pressure–flow information and cannot distinguish specific urodynamic mechanisms underlying neurogenic lower urinary tract dysfunction (e.g., detrusor overactivity, detrusor–sphincter dyssynergia, or impaired contractility with retention). We therefore describe an overflow incontinence–like voiding pattern as an interpretation based on convergent VSA features (leakage/tailing and altered spot morphology) together with gross evidence of bladder distension and increased bladder weight, while acknowledging that definitive classification would require cystometry (± EUS electromyography) and objective post-void residual quantification. For clarity, this terminology is used as an operational, descriptive analogy to convey an incomplete-emptying–type behavior (overdistended bladder with dribbling/leakage), rather than a urodynamically confirmed diagnosis. Moreover, because pressure–flow parameters and sphincter activity were not measured in the present study, we cannot determine whether detrusor overactivity, reflex/high-pressure voiding, or detrusor–sphincter dyssynergia contributed to the observed VSA patterns. Future studies incorporating longitudinal urodynamics, single-cell or spatial transcriptomics, and targeted interventions against specific AFI-axis members will be essential to clarify how this signaling axis contributes to the onset and progression of neurogenic bladder.

In summary, this study demonstrates that SCI-related overflow incontinence is accompanied by structured remodeling of the AFI axis in both the bladder and kidney, characterized by tissue- and compartment-specific upregulation of FST and INHBB and downregulation of INHA and INHBE. AFI-axis reprogramming thus emerges as a key molecular feature of neurogenic bladder and its associated upper tract adaptations. Components of the AFI axis, including FST and distinct inhibin/activin subunits, represent candidate mechanistic nodes and potential biomarkers, and their therapeutic relevance should be evaluated in future interventional studies with objective functional endpoints.

## Data Availability

The original contributions of this study are included in the article and its Supplementary Material. For further inquiries, please contact the corresponding authors.

## References

[B1] Almajid F. Kang D.-Y. Ahn J.-M. Park S.-J. Park D.-W. (2002). Urinary tract infection in patients with neurogenic bladder dysfunction. Int. Braz J. Urol. 19, 592–597. 10.1016/S0924-8579(02)00114-0 12135853

[B2] Almajid F. Kang D.-Y. Ahn J.-M. Park S.-J. Park D.-W. (2017). Long-term response of different botulinum toxins in refractory neurogenic detrusor overactivity due to spinal cord injury. Int. Braz J. Urol. 43, 721–729. 10.1590/s1677-5538.ibju.2016.0584 28537692 PMC5557449

[B3] Barrero J. A. Villamil Camargo L. M. Imaz J. N. Arciniegas Villa K. D. Rubio-Romero J. A. (2009). Maternal serum activin A, inhibin A and follistatin-related proteins across preeclampsia: insights into their role in pathogenesis and prediction. J. Mother Child. 27, 119–133. 10.34763/jmotherandchild.20232701.d-23-00002 PMC10438925 37595293

[B4] Bian X. Snow Z. K. Zinn C. J. Gowan C. C. Conley S. M. Bratulin A. L. (2025). Activin A antagonism with follistatin reduces kidney fibrosis, injury, and cellular senescence-associated inflammation in murine diabetic kidney disease. Kidney360 6, 1278–1291. 10.34067/KID.0000000776 40152935 PMC12407135

[B5] Bouzoni E. Perakakis N. Mantzoros C. S. (2020). Circulating profile of activin-follistatin-inhibin axis in women with hypothalamic amenorrhea in response to leptin treatment. Metabolism 113, 154392. 10.1016/j.metabol.2020.154392 33045195 PMC7680407

[B6] Bouzoni E. Gavriil E. Anastasilakis A. D. Dovas D. Joshi A. Briana D. D. (2022). Embryo quality may be associated with serum inhibin B levels but not with serum or follicular fluid levels of other components of the activin–follistatin–inhibin axis. Endocr. Pract. 28, 1086–1090. 10.1016/j.eprac.2022.08.001 35944841

[B7] Burns A. S. Rivas D. A. Ditunno J. F. (2001). The management of neurogenic bladder and sexual dysfunction after spinal cord injury. Spine (Phila Pa 1976) 43, S129–S136. 10.1097/00007632-200112151-00022 11805620

[B8] de Groat W. C. (1998). Anatomy of the central neural pathways controlling the lower urinary tract. Eur. Urol. 34, 2–5. 10.1159/000052265 9705544

[B9] De Jong F. H. (1988). Inhibin. Physiol. Rev. 68, 555–607. 10.1152/physrev.1988.68.2.555 3282246

[B10] Hashimoto M. Karnup S. Daugherty S. L. Cho K. J. Banno E. Shimizu N. (2024). Sex differences in lower urinary tract function in mice with or without spinal cord injury. Neurourol. Urodynamics 43, 267–275. 10.1002/nau.25323 37916422 PMC10872808

[B11] Hedger M. P. de Kretser D. M. (2013). The activins and their binding protein, follistatin—Diagnostic and therapeutic targets in inflammatory disease and fibrosis. Cytokine and Growth Factor Rev. 24 (3), 285–295. 10.1016/j.cytogfr.2013.03.003 23541927

[B12] Ma L. Mu Y. Li X. Zhang M. An W. Zeng F. (2023). Expression of transforming growth factor-β1 and autophagy markers in the bladder of rats with neurogenic lower urinary tract injury. Spinal Cord. 61, 154–159. 10.1038/s41393-022-00866-y 36319684

[B13] Mao W. Zhang T. Chen H. Barge S. Wang Z. Olumi A. (2025). Expression and distribution of activin-follistatin-inhibin axis in the urinary bladder. Front. Mol. Biosci. 12, 1519977. 10.3389/fmolb.2025.1519977 40144023 PMC11936821

[B14] Nagayama I. Takei Y. Takahashi S. Okada M. Maeshima A. (2025). The activin-follistatin system: key regulator of kidney development, regeneration, inflammation, and fibrosis. Cytokine and Growth Factor Rev. 81, 1–8. 10.1016/j.cytogfr.2024.11.004 39581798

[B15] Perakakis N. Upadhyay J. Ghaly W. Chen J. Chrysafi P. Anastasilakis A. D. (2018). Regulation of the activins-follistatins-inhibins axis by energy status: impact on reproductive function. Metabolism 85, 240–249. 10.1016/j.metabol.2018.05.003 29752954 PMC6062472

[B16] Peyronnet B. Mironska E. Chapple C. Cardozo L. Oelke M. Dmochowski R. (2019). A comprehensive review of overactive bladder pathophysiology: on the way to tailored treatment. Eur. Urol. 75, 988–1000. 10.1016/j.eururo.2019.02.038 30922690

[B17] Ryu J. C. Tooke K. Malley S. E. Soulas A. Weiss T. Ganesh N. (2018). Role of proNGF/p75 signaling in bladder dysfunction after spinal cord injury. J. Clin. Invest. 128, 1772–1786. 10.1172/JCI97837 29584618 PMC5919823

[B18] Tsai M. T. Ou S. M. Lee K. H. Lin C. C. Li S. Y. (2024). Circulating activin A, kidney fibrosis, and adverse events. Clin. J. Am. Soc. Nephrol. 19, 169–177. 10.2215/CJN.0000000000000365 37983094 PMC10861103

[B19] Tsuchida K. Nakatani M. Hitachi K. Uezumi A. Sunada Y. Ageta H. (2009). Activin signaling as an emerging target for therapeutic interventions. Cell. Commun. Signal 7, 15. 10.1186/1478-811X-7-15 19538713 PMC2713245

[B20] Wang N. Lu L. Cao Q. Qian S. Ding J. Wang C. (2021). Partial inhibition of activin receptor-like kinase 4 alleviates bladder fibrosis caused by bladder outlet obstruction. Exp. Cell. Res. 406, 112724. 10.1016/j.yexcr.2021.112724 34237300

[B21] Wang Y. Tang Q. Sun Q. Kopač M. Tsai M.-T. Ou S.-M. (2024). Pediatric lower urinary tract dysfunction: a comprehensive exploration of clinical implications and diagnostic strategies. Biomedicines 12, 945. 10.3390/biomedicines12050945 38790908 PMC11118197

[B22] Wang Y. Tang Q. Sun Q. (2025). INHBA knockdown inhibits renal fibrosis in mice following ischemia–reperfusion injury by suppressing activation of the TGF-β/Smad signaling pathway. BMC Nephrol. 26, 526. 10.1186/s12882-025-04443-2 41013408 PMC12465377

[B23] Wijayarathna R. de Kretser D. M. (2016). Activins in reproductive biology and beyond. Hum. Reproduction Update 22, 342–357. 10.1093/humupd/dmv058 26884470

[B24] World Health Organization (2024). Spinal Cord Injury. Available online at: https://www.who.int/news-room/fact-sheets/detail/spinal-cord-injury (Accessed January 11, 2026).

