# Supplementary figures and images for: Spinal cord injury–induced overflow incontinence reshapes the activin–follistatin–inhibin axis in mouse bladder and kidney
Source: Front Mol Biosci. 2026 Feb 18;13:1752395. doi: 10.3389/fmolb.2026.1752395 (PMC12957133; doi:10.3389/fmolb.2026.1752395)

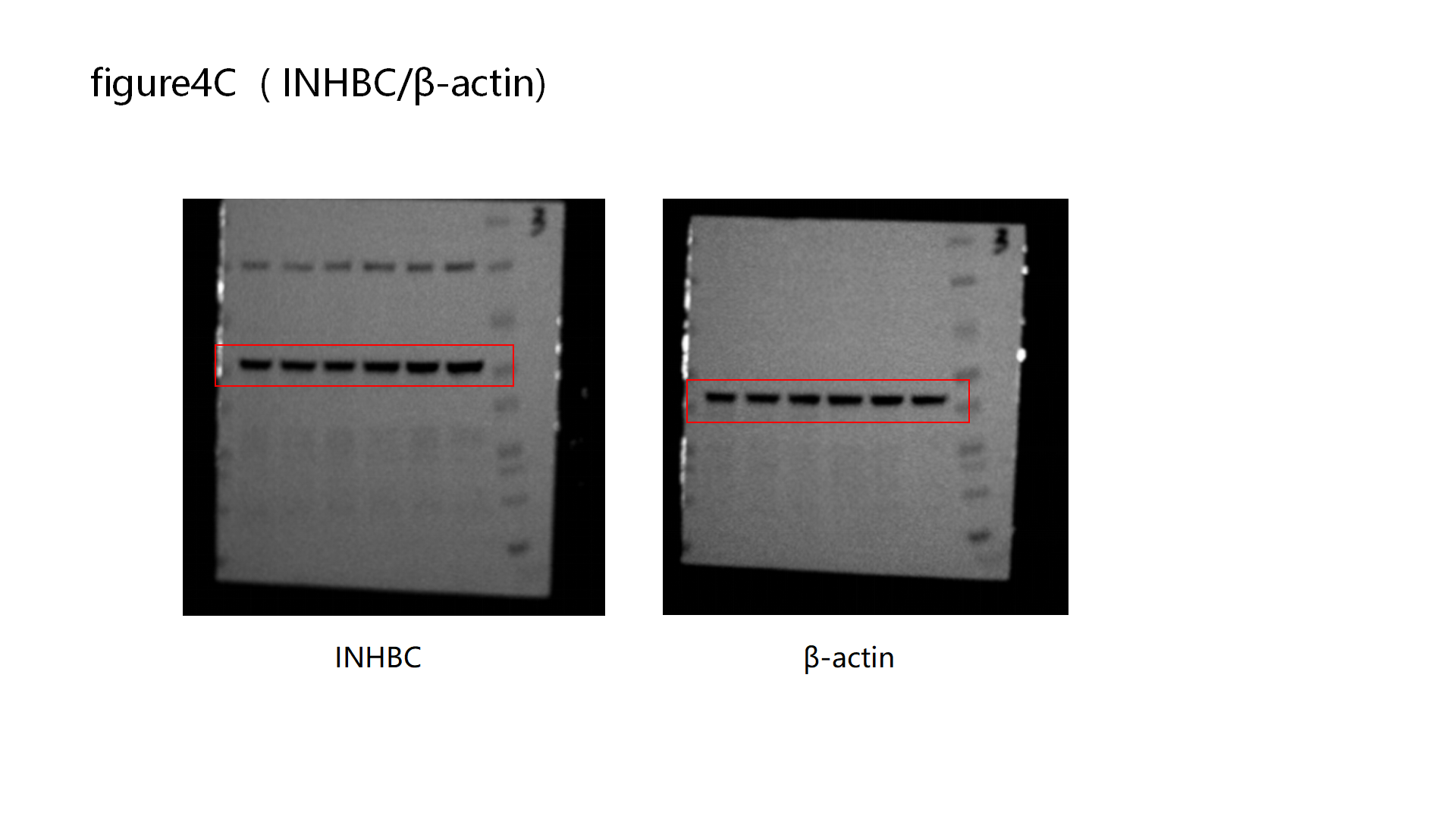

Supplement: Supplementary file 1 [file Image6.tif]

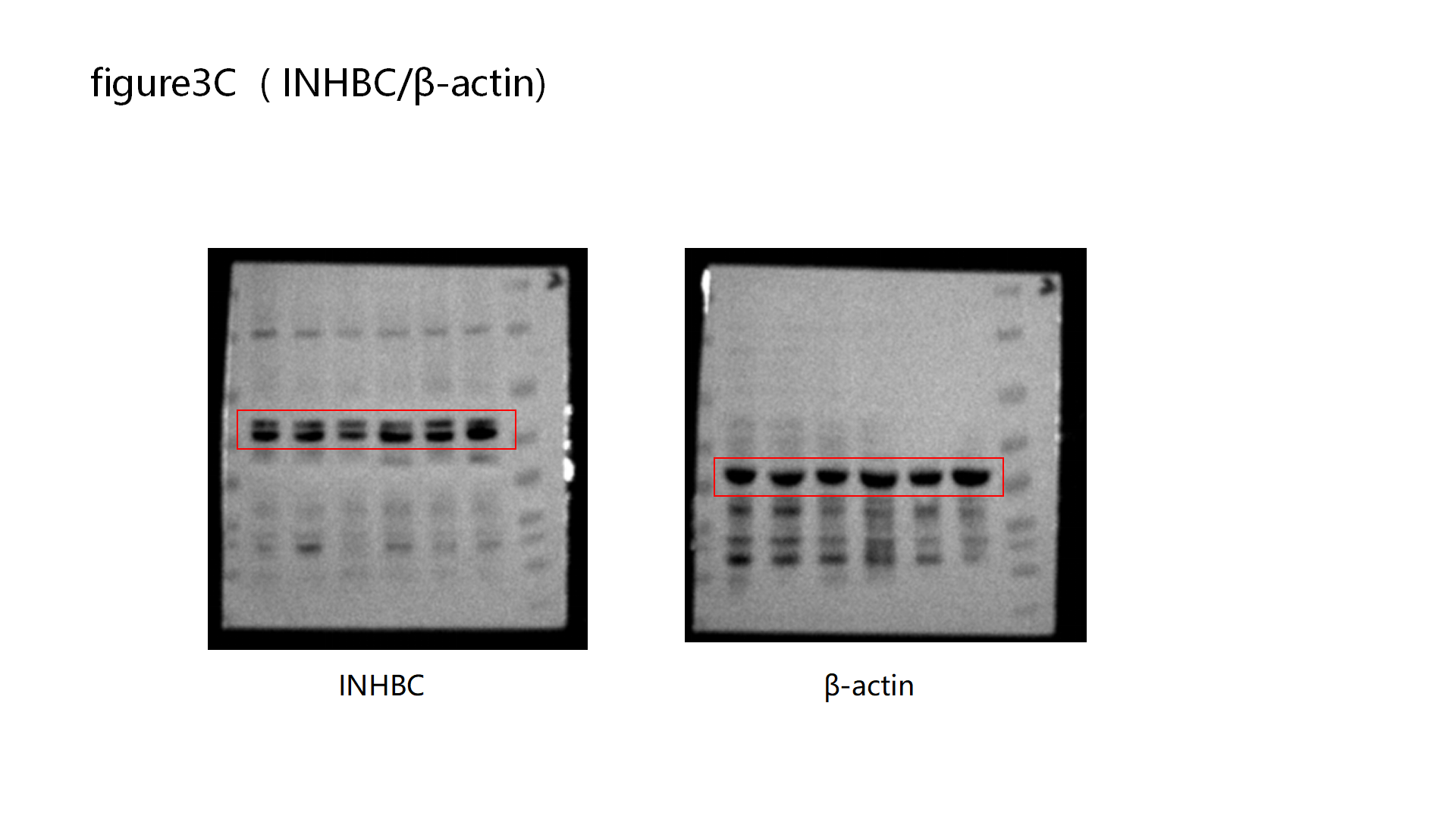

Supplement: Supplementary file 3 [file Image3.tif]

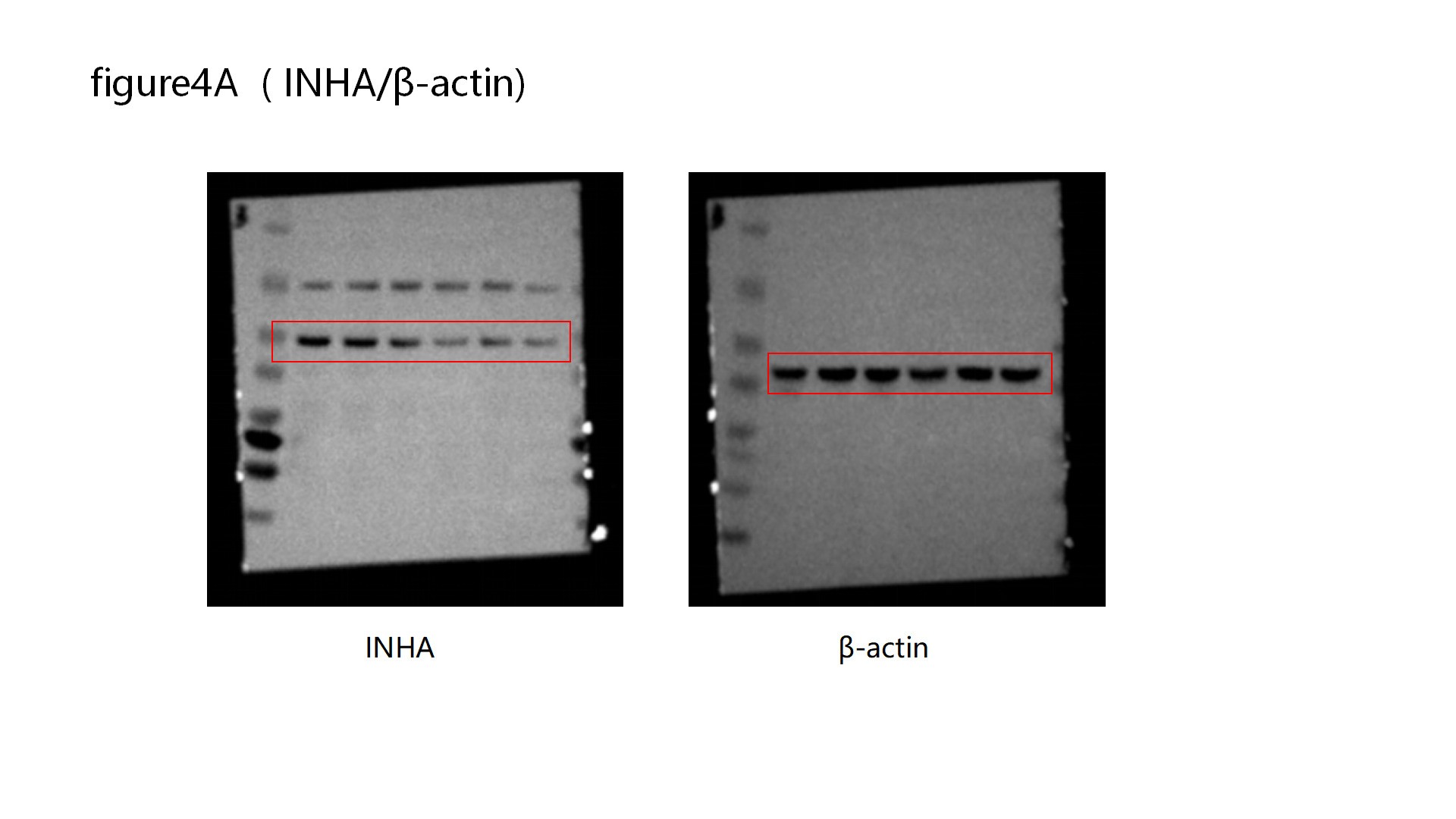

Supplement: Supplementary file 4 [file Image4.tif]

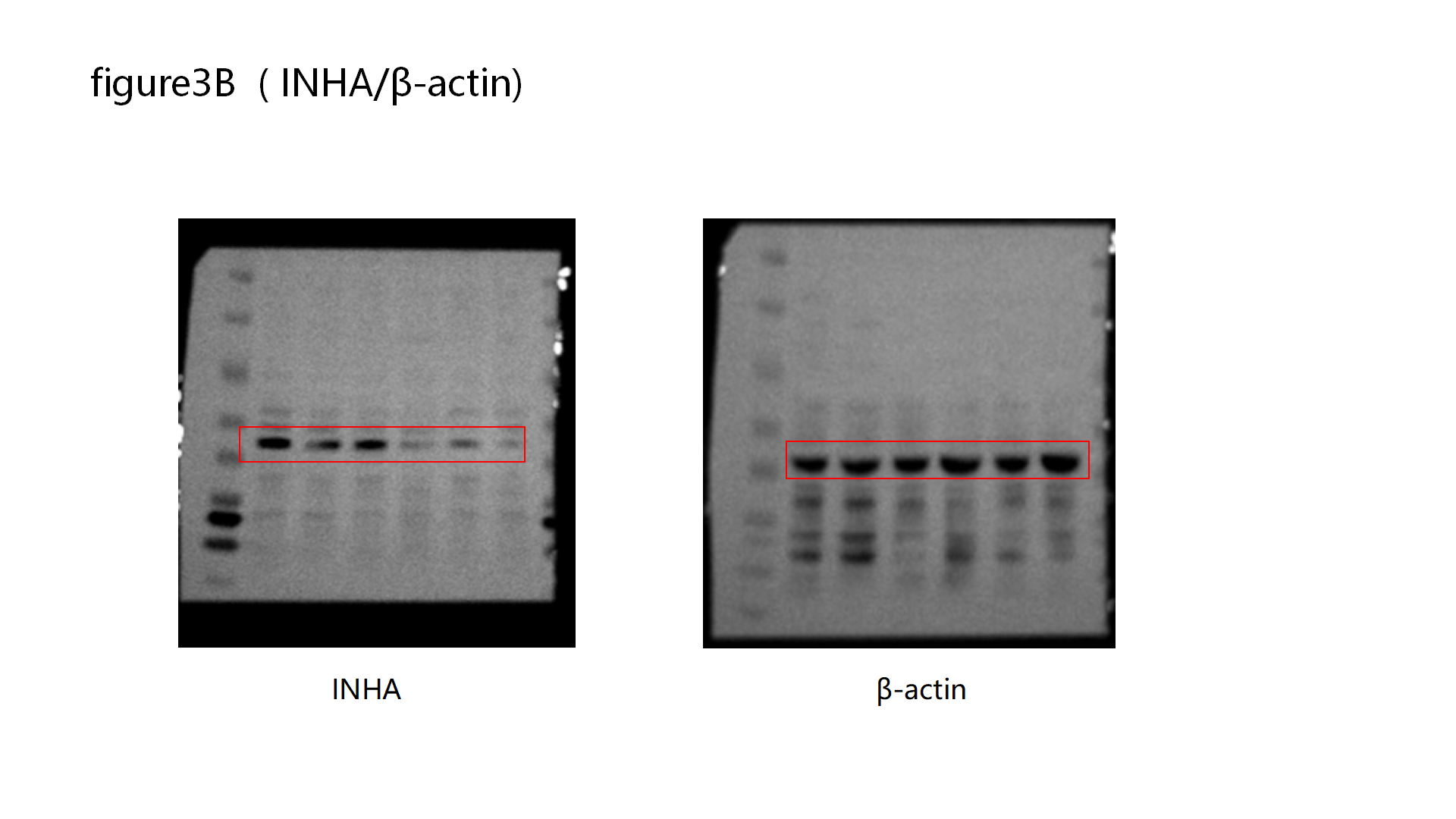

Supplement: Supplementary file 5 [file Image2.tif]

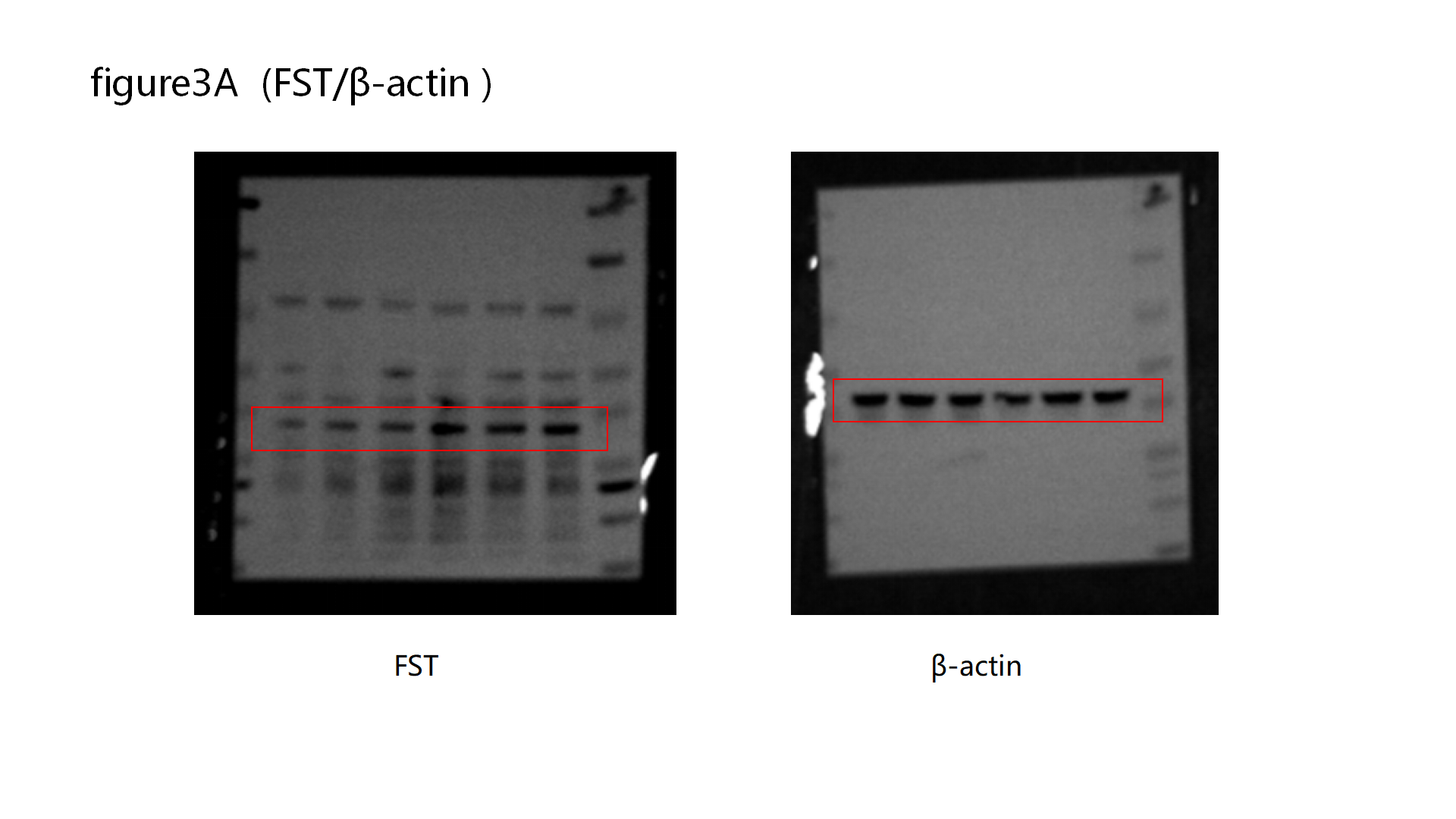

Supplement: Supplementary file 6 [file Image1.tif]

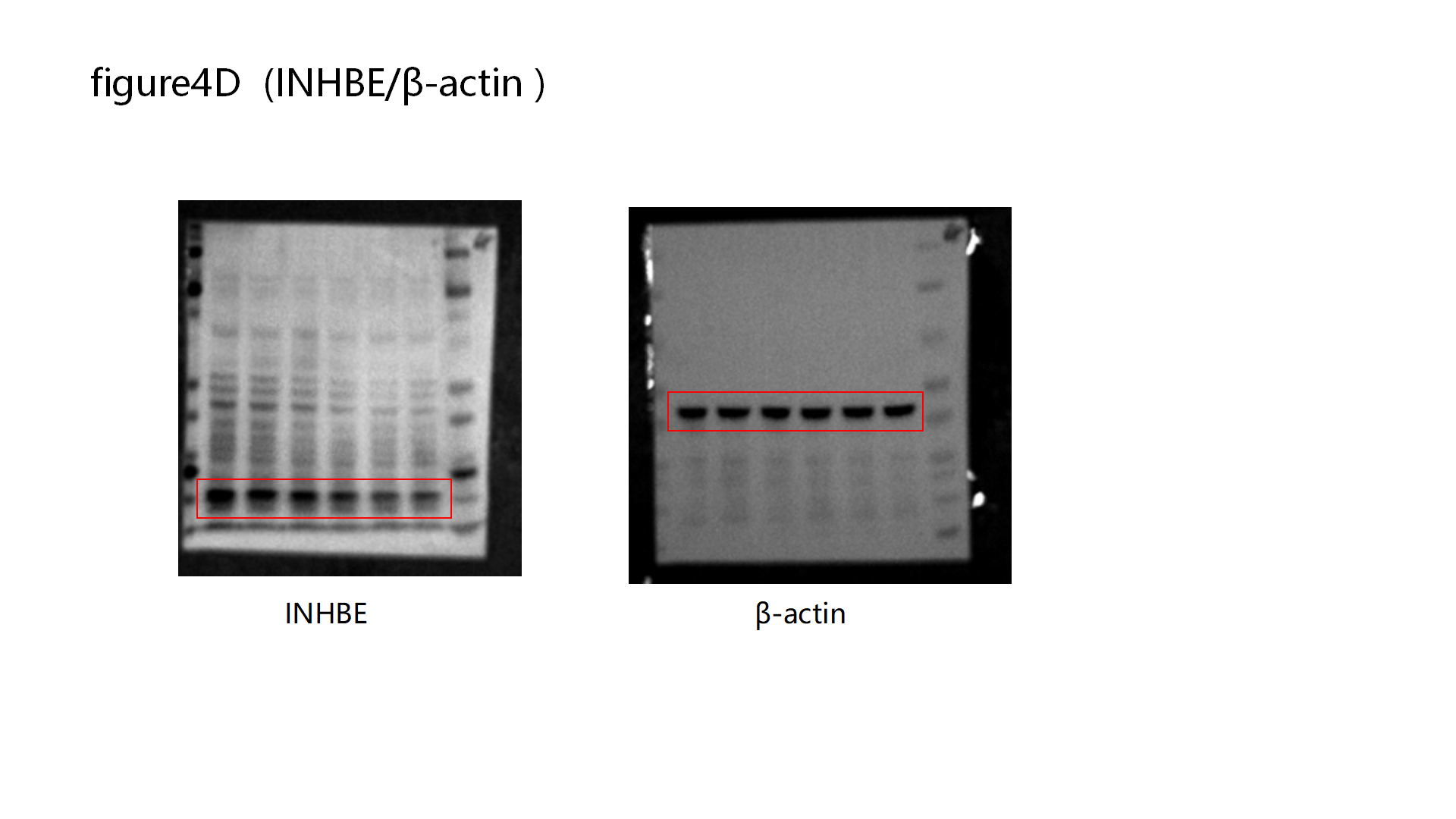

Supplement: Supplementary file 7 [file Image7.tif]

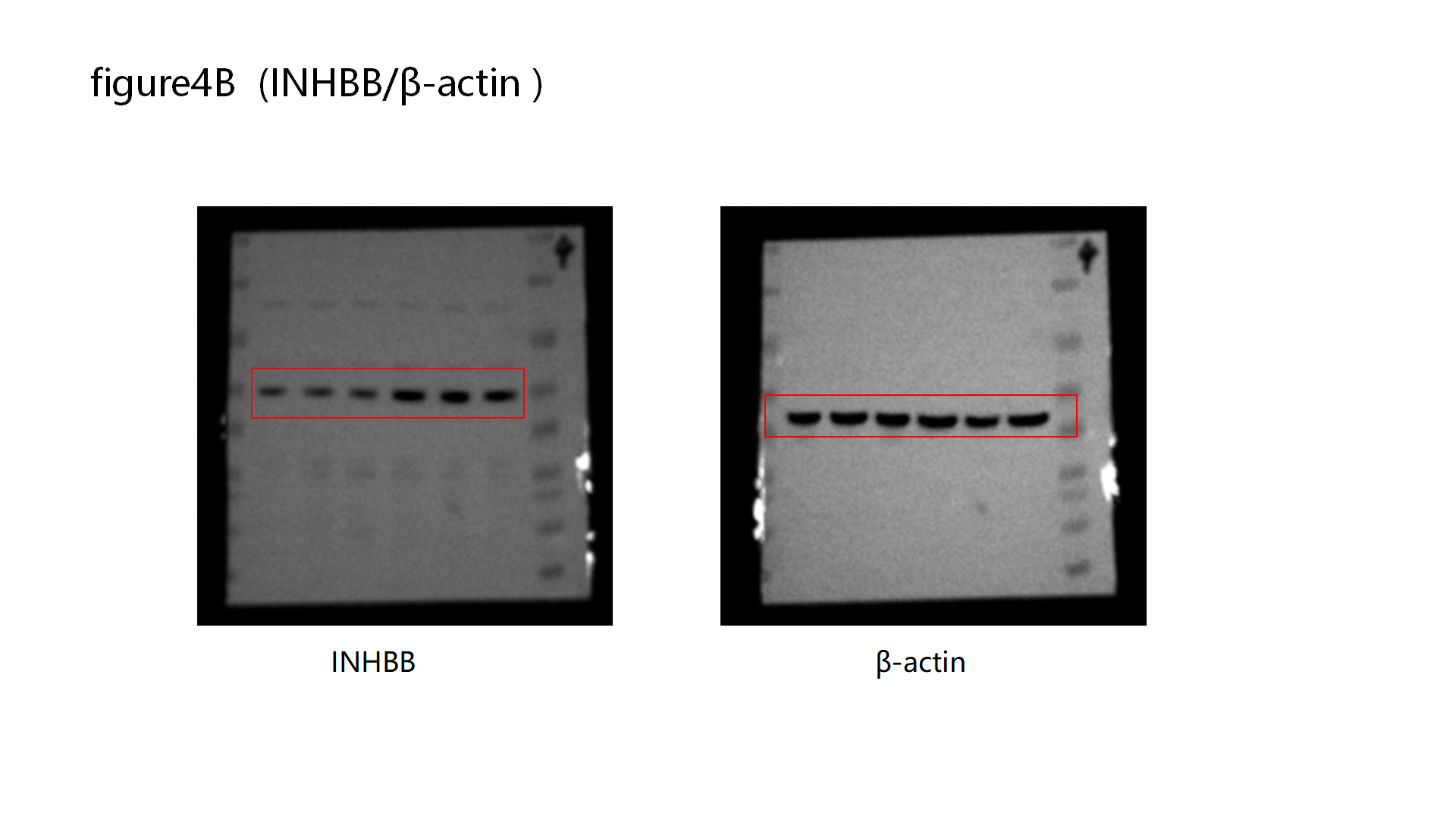

Supplement: Supplementary file 8 [file Image5.tif]
