# Supplementary material for: Spinal cord injury–induced overflow incontinence reshapes the activin–follistatin–inhibin axis in mouse bladder and kidney
Source: Front Mol Biosci. 2026 Feb 18;13:1752395. doi: 10.3389/fmolb.2026.1752395 (PMC12957133; doi:10.3389/fmolb.2026.1752395)
Supplement: Supplementary file 2 [file Supplementaryfile1.docx]

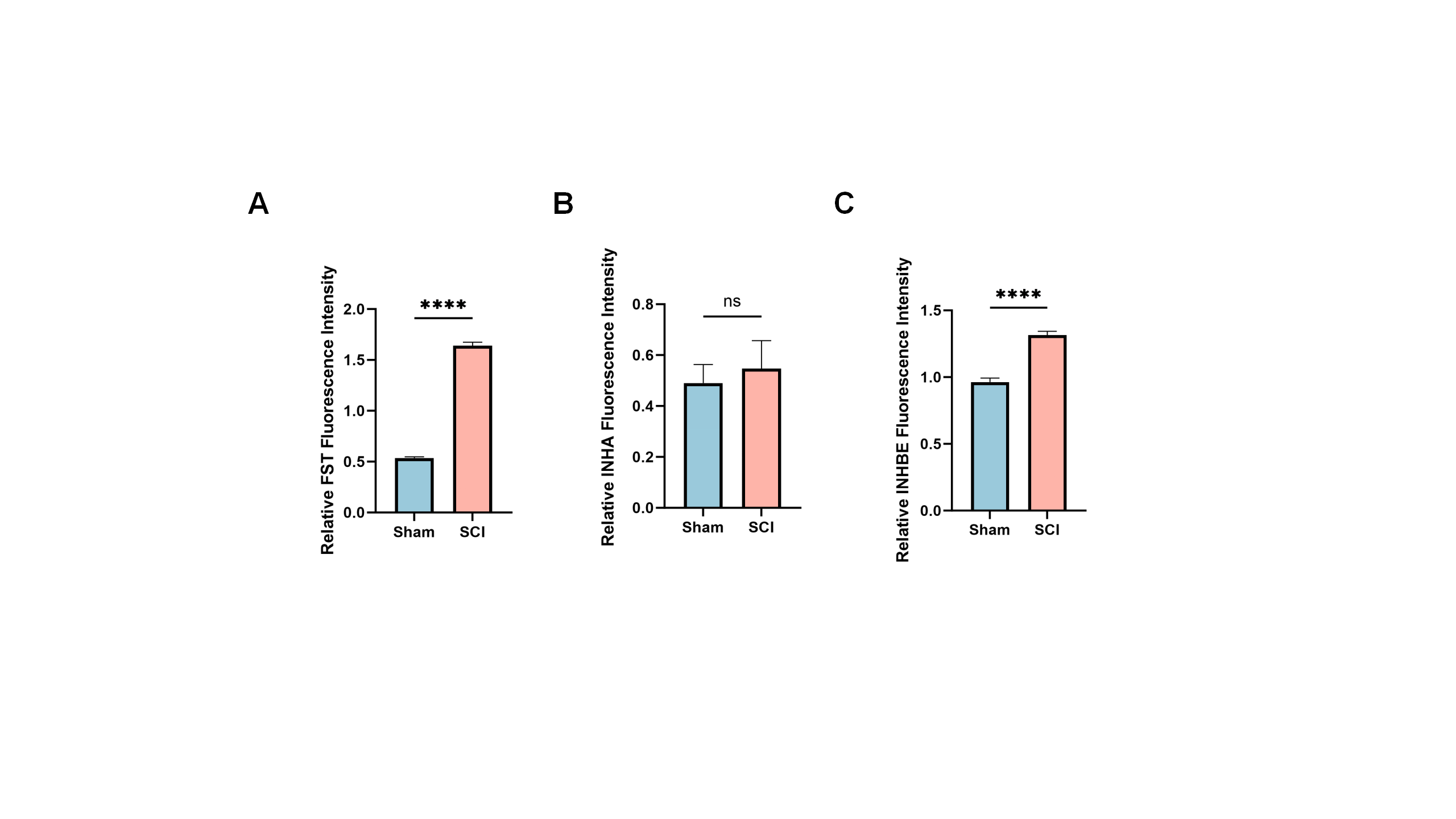


Figure S1. Semi-quantitative immunofluorescence analysis of AFI-axis proteins in the bladder after SCI.

Relative fluorescence intensity of FST (A), INHA (B), and INHBE (C) in bladder sections from Sham and SCI mice. Bars represent group means with error bars. Statistical significance is indicated as shown in the panels (ns, not significant; ****, P < 0.0001).


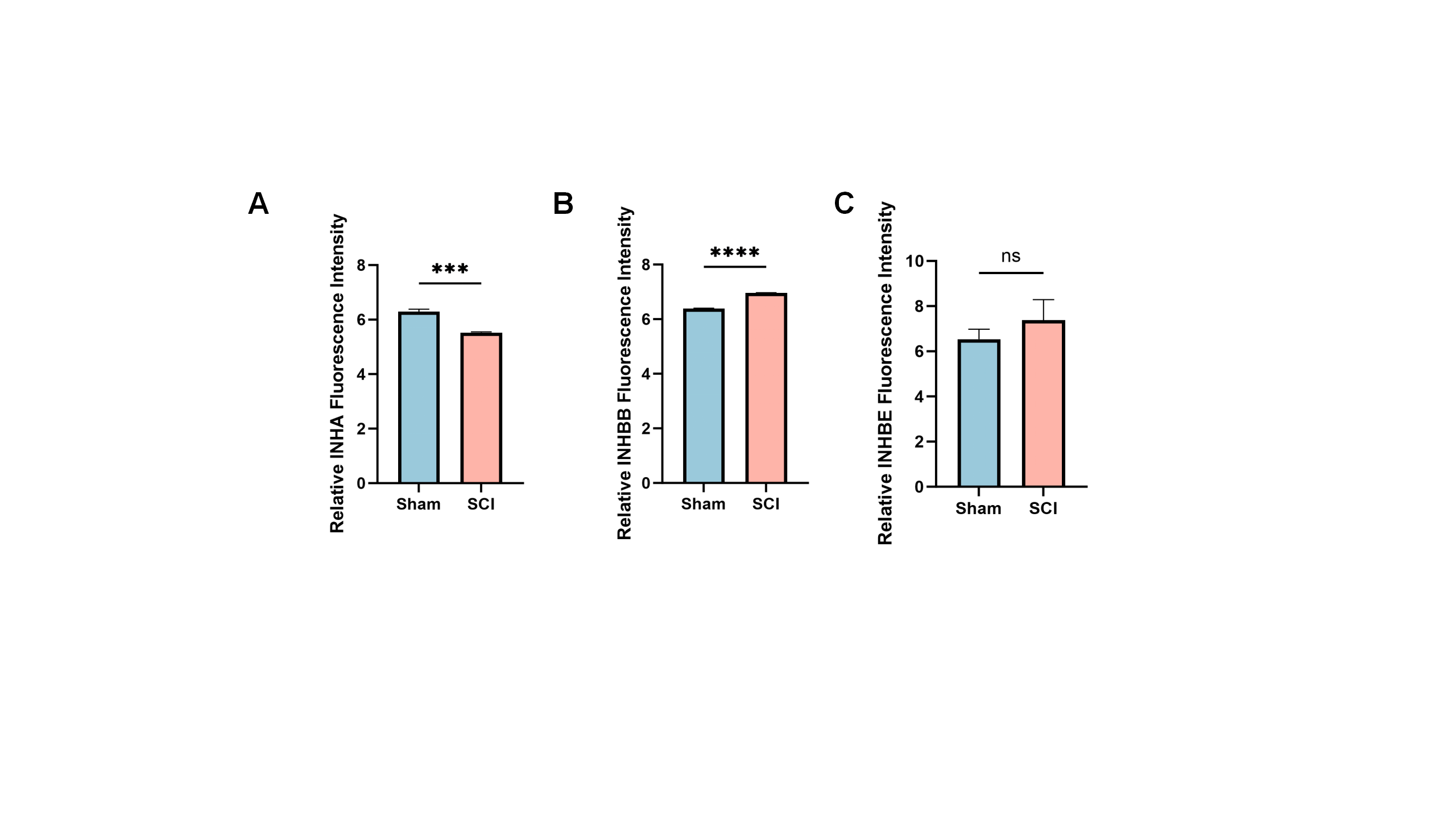


Figure S2. Semi-quantitative immunofluorescence analysis of AFI-axis proteins in the kidney after SCI.
Relative fluorescence intensity of INHA (A), INHBB (B), and INHBE (C) in kidney sections from Sham and SCI mice. Bars represent group means with error bars. Statistical significance is indicated as shown in the panels (ns, not significant; ***, P < 0.001; ****, P < 0.0001).
